# Sulphur and carbon isotopes as tracers of past sub-seafloor microbial activity

**DOI:** 10.1038/s41598-018-36943-7

**Published:** 2019-01-24

**Authors:** Patrick Meister, Benjamin Brunner, Aude Picard, Michael E. Böttcher, Bo Barker Jørgensen

**Affiliations:** 1 Department of Geodynamics and Sedimentology, University of Vienna, Althanstr. 14, 1090 Vienna, Austria; 2 Department of Geological Sciences, University of Texas at El Paso (UTEP), El Paso, TX 85287-1404 USA; 3 School of Life Sciences, University of Nevada, Las Vegas, 4505 S. Maryland Parkway, Las Vegas, NV 89154-4004 USA; 4 Geochemistry & Isotope Biogeochemistry Group, Leibniz-Institute for Baltic Sea Research (IOW), Seestrasse 15, D-18119 Warnemünde, Germany; 5 Center for Geomicrobiology, Aarhus University, Ny Munkegade 114-116, 8000 Aarhus, Denmark; 6 Max-Planck Institute for Marine Microbiology, Celsiusstrasse 1, D-28359 Bremen, Germany

## Abstract

Microbial life below the seafloor has changed over geological time, but these changes are often not obvious, as they are not recorded in the sediment. Sulphur (S) isotope values in pyrite extracted from a Plio- to Holocene sequence of the Peru Margin (Ocean Drilling Program, ODP, Site 1229) show a down-core pattern that correlates with the pattern of carbon (C) isotopes in diagenetic dolomite. Early formation of the pyrite is indicated by the mineralogical composition of iron, showing a high degree of pyritization throughout the sedimentary sequence. Hence, the S-record could not have been substantially overprinted by later pyrite formation. The S- and C-isotope profiles show, thus, evidence for two episodes of enhanced microbial methane production with a very shallow sulphate-methane transition zone. The events of high activity are correlated with zones of elevated organic C content in the stratigraphic sequence. Our results demonstrate how isotopic signatures preserved in diagenetic mineral phases provide information on changes of past biogeochemical activity in a dynamic sub-seafloor biosphere.

## Introduction

The exploration of the sub-seafloor biosphere over the last two decades has provided detailed information on microbial distribution, metabolic activity, and subsurface redox zonation (Parkes *et al*.^1^; D’Hondt *et al*.^2,3^; Jørgensen *et al*.^4^; Kallmeyer and Wagner^5^). While the ongoing processes are now relatively well understood, it remains poorly assessed how the microbial activity responded to past oceanographic, palaeo-climatic, and depositional conditions. Large changes in sub-surface microbial activity occur over timescales of 100,000 years or more and these are triggered by oceanographic variations, such as glacial-interglacial cycles (Aiello and Bekins^6^; Contreras *et al*.^7^; Meister^8^). Resulting changes in subsurface geochemical conditions cannot simply be traced by the present downcore distribution of microbial activity or by the modern porewater chemistry. Instead, tracing variations of deep biosphere conditions over geological time relies on proxies that are preserved in the solid-phase diagenetic record for millions of years (e.g. Kelts and McKenzie^9^; Meister *et al*.^10^; Schrag *et al*.^11^). Elemental and isotopic signatures may be preserved if they are enclosed inside of diagenetic mineral phases, ideally, if they are partitioned in the crystal lattice. Diagenetic minerals may themselves be the product of specific microbial processes, and information on these processes becomes permanently trapped at the time of precipitation.

A range of different mineral archives, such as dolomite, siderite, rhodochrosite or quartz, has been suggested as indicators of specific metabolic pathways (as compiled in Meister^8^). Furthermore, several isotope systems are indicative of microbial activity (e.g. Kaplan and Rittenberg^12^; Claypool and Kaplan^13^; Jørgensen^14^). Specifically, light stable isotopes bear the advantage that they show large isotope variations mostly considered to result from kinetic fractionation effects of enzymatically-controlled reactions. Particularly large isotope fractionation is observed for S isotopes between oxidized and reduced S (e.g., Hoefs^15^). Recently, Parnell *et al*.^16^ observed that S isotopes are excellent markers for past microbial activity in sand injections that occur within long records of continental margin deposits. This insight is intriguing as S isotope records are available in many organic carbon-rich sedimentary sequences, providing potential archives of past sub-seafloor microbial activity.

The S isotope composition (δ^34^S, a measure for the deviation of the isotope ratio of a sample from the international reference Vienna Canyon Diablo Troilite, VCDT, which for convenience is reported in ‰ values) of sulphate and sulphide is a good indicator for sulphate-reducing activity. Sulphide produced during microbial sulphate reduction is depleted in ^34^S relative to the sulphate, with an S isotope discrimination, referred to as S isotope enrichment factor (ε^34^S) of up to 75‰ (e.g., Sim *et al*.^17^). Accordingly, the residual sulphate is increasingly enriched in ^34^S as more sulphate is consumed. The δ^34^S values in porewater sulphate thus show an increase with depth in the sulphate reduction zone, and the isotopic composition of the produced sulphide follows the same trend but is offset, approximately by the value of the apparent ε^34^S in the porewater (Jørgensen^14^; Hartmann and Nielsen^18^). Near the sulphate methane transition zone (SMTZ), where sulphate is almost entirely consumed, the δ^34^S of sulphide can therefore reach very high values (e.g. Borowski *et al*.^19^; Pellerin *et al*.^20^). As sulphate is the essential electron acceptor in anoxic sediments, and the sulphate reduction rate is stoichiometrically coupled to the organic carbon mineralization rate, the gradient at which δ^34^S increases with depth is also indicative of the overall dissimilatory activity in anoxic sediments. We propose that this increase is reflected in the δ^34^S of pyrite.

The challenge lies in the understanding of how this signal is recorded, because the δ^34^S of pyrite from a sediment sample averages all δ^34^S values of individual pyrite grains that accumulated over time within the zone of pyrite formation. The depth of the pyrite formation zone depends on the availability of reactive iron, modified by the dynamics of the sedimentary system. Moreover, existing records can be overprinted or even erased by subsequent biological or abiotic processes. Such overprinting may particularly occur where methanogenic zones persist at shallow depth for a very long time, so that under sulphide-free conditions substantial re-distribution of Fe may take place (e.g. Riedinger *et al*.^21^).

**Figure 1:**
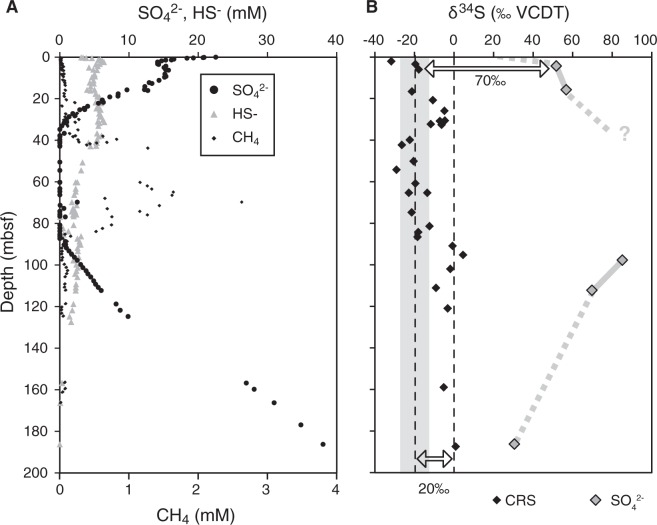
Porewater chemistry and S isotope data from ODP Site 1229. (**A**) Sulphate, sulphide and methane concentrations in porewater extracted from drill cores at ODP Site 1229 (D’Hondt *et al*.^2^). (**B**) Sulphur isotope values in chromium-reducible sulphide. Sulphur isotope values in dissolved sulphate from Böttcher *et al*.^21^ are plotted for comparison.

We present a profile of δ^34^S-values extracted from diagenetic pyrite through a 200-m-thick sediment sequence at ODP Site 1229 to assess its validity as an archive of microbial activity through the last >2 Ma. This site shows a sulphate depletion through the top 30 m below seafloor (mbsf) with a SMTZ at 35 mbsf and a second SMTZ at 90 mbsf, below which sulphate diffuses upwards from a deep-seated brine (D’Hondt *et al*.^2^; Fig. 1A; further information on the geological setting is provided as Supplementary Material). Dissolved total sulphide (here referred to as HS^−^) was detected throughout the entire sediment column, with near zero concentrations around 75 to 95 mbsf, and at the bottom of the retrieved sediment (D’Hondt *et al*.^2^). To test if pyrite was formed early after deposition or at a later stage, the amount of iron (Fe) bound to pyrite was quantified via the content of chromium-reducible S (CRS) and compared to the content of Fe-fractions showing different reactivity in the sediment. The Fe-fractions were determined using a sequential iron extraction procedure combined with Fe K-edge X-ray absorption near-edge structure (XANES) spectroscopy of iron minerals (three samples from different depths). Additional insights into coupled Fe-S cycling were gained by the comparison of the δ^34^S of modern-day dissolved sulphate in the porewater to the δ^34^S of pyrite. The Fe-S record was then juxtaposed with the total organic carbon (TOC) content and a previously measured δ^13^C record in diagenetic dolomite, to identify a potential co-variation that would be indicative of changes in microbial activity over time.

## Results and Discussion

### The S isotope record in pyrite

The downcore distribution of δ^34^S values in pyrite (CRS; Fig. 1B; Table S1) shows values between −30‰ and +5‰. In the top few metres of the sedimentary sequence, pyrite shows δ^34^S values around −20‰, and similar values are found in the middle part, within the present-day methanogenic zone. Higher values, reaching 0‰ or more occur near 30 m and 100 m below seafloor (mbsf), and one value near 180 mbsf. The isotope offset of about 70‰ between pyrite and sulphate in porewater of the uppermost few metres (data from Böttcher *et al*.^22^) is at the upper limit of fractionation by microbial sulphate reduction commonly observed in marine sediments (e.g. Wortmann *et al*.^23^; Jørgensen *et al*.^24^; Pellerin *et al*.^20^). The offset increases to 80‰ around 100 mbsf but decreases to about 30‰ near the bottom of the sequence, hence showing a strong variation in the apparent fractionation. The highly variable offset between the δ^34^S signatures of pyrite and sulphate is not consistent with pyrite precipitation from present porewater sulphide, which would expectedly have a more constant offset from porewater sulphate (cf. Jørgensen *et al*.^24^). Several factors could have influenced the profile of δ^34^S in pyrite, such as (1) the depth of pyrite formation, (2) variations of ε^34^S, (3) changes in microbial activity, or (4) sedimentation rate. Here we discuss which factors are relevant and whether δ^34^S in pyrite may indeed record overall dissimilatory activity in ancient sediments.

### The depth of pyrite formation

**Figure 2:**
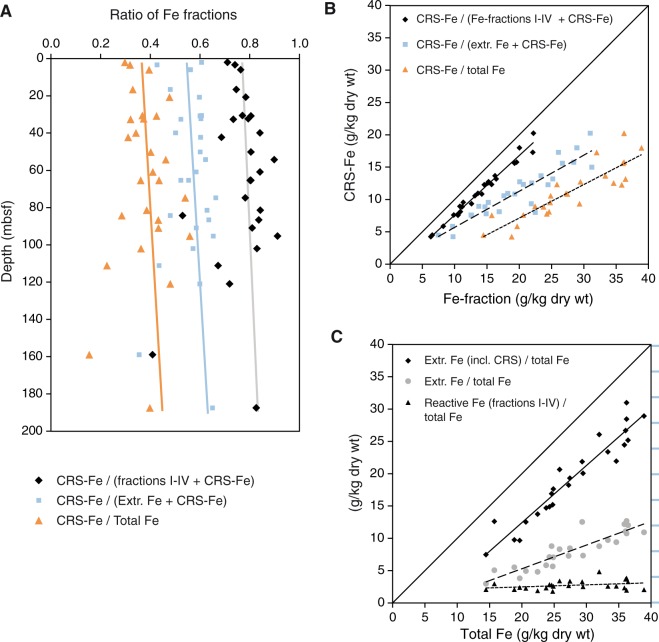
Abundance of pyrite-bound iron relative to different solid-phase iron fractions in sediment of ODP Site 1229. (**A**) Degree of pyritization with respect to total Fe (orange triangles), extracted Fe (light-blue squares), and extracted Fe without the poorly reactive silicate fraction (black diamonds) plotted vs. depth. (**B**) Cross-plot of CRS-bound Fe (pyrite) vs. total Fe, extracted Fe + CRS-Fe, and extracted Fe + CRS-Fe without the poorly reactive silicate fraction. (**C**) Plot of reactive Fe vs. different Fe-fractions to show extraction efficiency. Lines shown in the plots are regression lines.

Ratios of CRS-related Fe to CRS-Fe + total sequentially extracted Fe (using the five-step extraction scheme of Poulton and Canfield^25^; see method description below) increase from the sediment water interface to ~0.6 within the top 2 mbsf and remain near this level through the profile (Fig. 2A,B; Table S1). These ratios approximately correspond to the degree of pyritization (DOP; ratio of pyritic Fe to pyritic Fe + acid-soluble Fe; Berner^26^), indicating that most pyrite forms within the uppermost 2 mbsf. The increase is even higher, to ~0.8, if only fractions I through IV of the extraction scheme are considered for calculation. According to Poulton and Canfield^25^ fractions I through IV essentially include all Fe-phases but the poorly reactive silicates. As shown by leaching experiments (Kasina *et al*.^27^) fraction III includes reactive sheet silicates, such as smectites, from which Fe could potentially react with sulphide.

**Figure 3:**
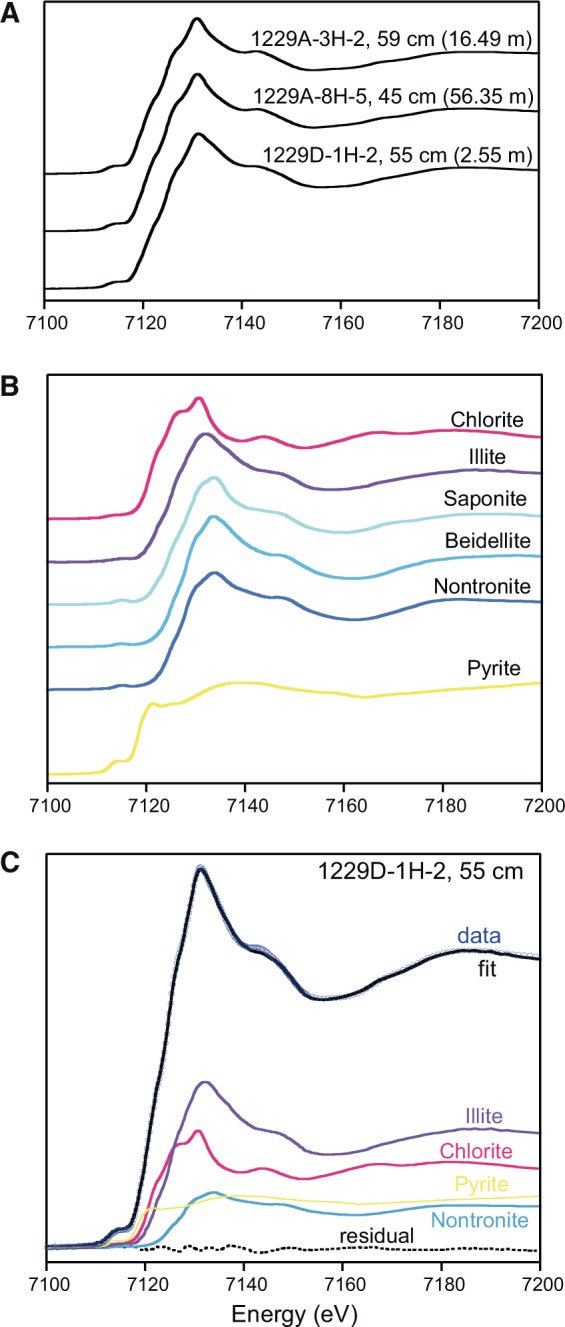
Fe K-edge XANES spectroscopy of sediments. (**A**) XANES spectra of sediment samples collected at ODP Site 1229 at 2.55, 16.49, and 56.35 mbsf. (**B**) XANES spectra of reference minerals of relevance for this study: sheet silicate minerals of the chlorite group, illite group and smectite group (beidellite, saponite, nontronite), and pyrite. (**C**) Example of fitting analysis for the sample at 2.55 mbsf that contains Fe in all three sheet silicate groups and in pyrite.

Fe K-edge XANES spectroscopy showed that Fe occurs in significant proportions as pyrite (17–26%), while the rest of the Fe is present in the structure of sheet silicates, mostly from the illite and the chlorite groups (Fig. 3). Only in the uppermost sample at 2.55 mbsf, some Fe was present in smectite. As XANES spectra at the Fe K-edge are similar for different smectites (Fig. 3B; Rennert *et al*.^28^), the contribution of the standard spectra we had available (beidellite, saponite, and nontronite) may also represent montmorillonite, which commonly occurs in marine sediments. Overall, the pyritizable Fe-fractions make up a minor amount of the total Fe in the sediment (triangles in Fig. 2C).

More abundant is the Fe bound in poorly reactive sheet silicates, which are extracted with boiling HCl (extraction step V). This fraction contributes up to 30% of the total Fe as visualized by the slope of the regression line of the total extracted Fe (fractions I–V) vs. total Fe in Fig. 2C (dashed line). Canfield^29^ has shown that the poorly reactive sheet silicates, such as chlorite and illite do not significantly react if exposed to sulphidic conditions over millions of years. Indeed, XANES analyses showed chlorite and illite as the most abundant Fe-mineral phases. The presence of these unreactive silicate phases explains why the ratio of CRS-Fe to total extracted Fe is only around 0.6 (blue regression line in Fig. 2A). An overestimation of CRS-Fe due to organically bound S is unlikely, as organic S has been shown to be largely non-extractable by Cr(II)-solution in Peru Margin sediments (Mossmann *et al*.^30^; cf. also Fossing and Jørgensen^31^), and would cause a perturbation of the linear trend of extracted vs. total Fe (Fig. 2D; cf. Böttcher *et al*.^32^). Because of the approximate extraction efficiency of 80% for sequential leaching of Fe (cf. Fig. 2C), the ratio of CRS-Fe to the total Fe-content, measured by X-ray fluorescence (orange regression line in Fig. 2A), is somewhat lower than the ratio of CRS-Fe to extracted Fe. Nevertheless, the same pattern is observed as for the ratio of CRS-Fe to extracted Fe (both with and without unreactive sheet silicates) and the regression lines of all three ratios in Fig. 2A show the same slope. The pool of reactive iron is exhausted in the uppermost few metres (~5 m) below the seafloor, and pyritizable Fe-fractions only make up a minor proportion of the total Fe in the rest of the sediment column.

The precipitation of Fe-sulphides is the result of reductive dissolution of reactive Fe(III) phases by free sulphide. HS^−^ is produced by microbial sulphate reduction and reacts abiotically with different reactive Fe-oxides and oxyhydroxides (Berner^26^; Rickard and Luther III^33^). The actual electron transfer from HS^−^ to the Fe(III) has been demonstrated to control rates of iron reduction, whereas binding of HS^−^ on the Fe-mineral surface is not rate limiting (Afonso and Stumm^34^). The reductive dissolution of Fe minerals is therefore not dependent on the HS^−^ concentration in the porewater, as long as HS^−^ is available. While Fe-sulphide (FeS) can be directly precipitated from the porefluid under sulphidic conditions, the formation of pyrite (FeS_2_) requires an additional oxidation step. According to Wächtershäuser^35^ and Thiel *et al*.^36^, the oxidation step may be coupled to the reduction of water to H_2_ (eq. 1), which, however, may be readily consumed if sulphate is present.

1 $${\rm{FeS}}+{{\rm{HS}}}^{-}+{{\rm{H}}}^{+}\to {{\rm{FeS}}}_{2}+{{\rm{H}}}_{2}$$

Consistent with this reaction, Riedinger *et al*.^37^ showed that acid volatile sulphide is rapidly converted to CRS if measurable quantities of free sulphide are present in the porewater. Within the sulphidic zone, the depth at which pyrite forms is, thus, limited and controlled by the amount and reactivity of solid-phase Fe(III). Based on the present activity at depth, where sulphide is still produced, it is reasonable to assume that sulphide was always available during the deposition of the 200-m-thick sediment sequence at ODP Site 1229, except, perhaps interrupted by episodes of expanded methanogenic zones, which however, did not cause a significant re-distribution of Fe. This is supported by the observation that the DOP increases rapidly in the uppermost sediment and remains rather constant through the sequence, suggesting that the reactive Fe-phases are rapidly pyritized in the uppermost few metres, independent of how intense the sulphate reduction rates are.

Therefore, δ^34^S in diagenetic pyrite at ODP Site 1229 reflects the conditions in the near surface porefluid, unless sediment was affected by a permanently shallow SMTZ (i.e. shallower than the 2.5 m that may have been episodically reached; Contreras *et al*.^7^) or the sediment was largely exposed to suboxic, i.e. sulphide-free conditions. This means that the pyrite has recorded the isotopic composition of dissolved sulphide in the top few metres of the sediment through time, and the sulphide produced at depth today is not preserved in the pyrite record. Assuming that the sedimentation rate was constant, the S isotope profile could thus be used as a record of average microbial sulphate-reducing activity in the near-surface sediment interval.

### Sulphur isotope fractionation between sulphate and sulphide

A high sulphate reduction activity in the top interval of sediment leads to a steep depth-gradient in δ^34^S (i.e. sulphide is more strongly enriched in ^34^S in the same depth interval), whereas a low sulphate reduction activity leads to a low depth-gradient in δ^34^S. Thus, if Fe-sulphide formation occurs over a constant depth range, one would observe less negative/more positive values preserved in the Fe-sulphides for situations in which the sulphate reduction activity was intense, i.e. higher δ^34^S values indicate higher sulphate reduction activity.

The situation is complicated by the fact that the sedimentation rate may change (Hartmann and Nielsen^38^; Pasquier *et al*.^39^). An increase in sedimentation rate would result in a larger depth range over which the Fe-sulphides form. At the same time, a higher sedimentation rate would lead to a more rapid burial of reactive organic matter, higher sulphate-reducing activity and accordingly steeper sulphate gradients (Meister *et al*.^40^). Indeed, an upward shift of the SMTZ could be shown for the Peru margin ODP Site 1229, where the age model in Contreras *et al*.^7^ suggests variations in sedimentation rate between 0.01 and 0.8 m/ka. The steeper gradient in δ^34^S would then also lead to more positive values in the Fe-sulphides.

A further uncertainty could be the isotope enrichment factor. However, large fluctuations in the ε^34^S are not expected for marine sediments that receive organic matter that – with regard to its composition and content – does not vary substantially over time. In laboratory experiments with sulphate reducing bacteria, ε^34^S increases when cell-specific sulphate reduction rates become smaller, but this effect is tied to the substrate, respectively the energy yield rather than the total availability of the substrate (e.g., Kaplan & Rittenberg^12^; Chambers & Trudinger^41^; Sim *et al*.^17^; Wing and Halevy^42^). One exception to that rule is sulphate reduction coupled to the anaerobic oxidation of methane (AOM), where ε^34^S depends on methane partial pressures and low methane availability at the SMTZ results in a large ε^34^S near 70‰ (Deusner *et al*.^43^). Such large ε^34^S values also are common for organoclastic sulphate reduction in marine sediments (Rudnicki *et al*.^44^; Wortmann *et al*.^23^; Claypool^45^; Sim *et al*.^17^; Pellerin *et al*.^20^), and in very shallow sediments (a few 10’s of cm) the observed ε^34^S can be amplified by disproportionation of S compounds tied to oxidative S-cycling (Canfield and Thamdrup^46^; Cypionka *et al*.^47^; Habicht *et al*.^48^). Thus, the isotopic difference between sulphate and sulphide of ~70‰ in the top few mbsf at Site 1229 could reflect a long-term average ε^34^S between sulphate and sulphide observed in many porewaters of marine sediments (e.g. Claypool^45^; Jørgensen *et al*.^24^; Böttcher *et al*.^22^). This offset should be representative for the true separation factor near the zone of pyrite formation. Thus, changes by more than 20‰ in the δ^34^S record of pyrite at Site 1229 are likely reflecting long-term shifts in the microbial activity in the top metres of the sub-seafloor.

### The diagenetic history at Peru Margin Site 1229

**Figure 4:**
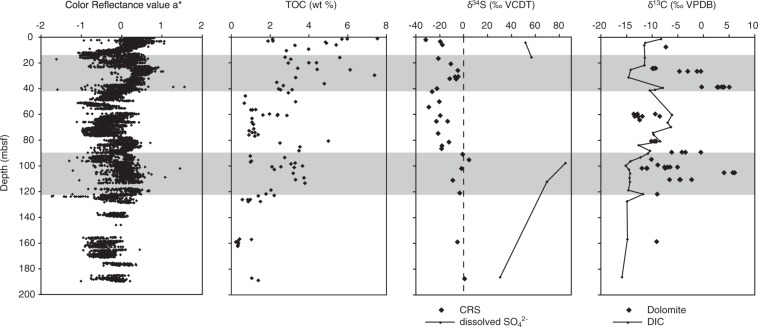
Correlation of δ^34^S in CRS with color reflectance values a*, TOC, and δ^13^C of diagenetic dolomites from ODP Site 1229. Color reflectance data are reproduced from (D’Hondt, *et al*.^2^); TOC data are compiled from Meister *et al*.^42^ and new data); δ^13^C of dissolved inorganic carbon and dolomite are from Meister *et al*.^10^.

For the reconstruction of the diagenetic history, the δ^34^S record of pyrite can be compared to several other parameters (Fig. 4). The two intervals showing higher δ^34^S-values also show somewhat elevated concentrations of total organic carbon (TOC). While the TOC data show a large scatter, and the measurements are not available at high resolution, shipboard core scans of the chromaticity value a* (red-green value; D’Hondt *et al*.^2^) have been shown to correlate with the abundance of diatom ooze (Meister *et al*.^49^; Aiello and Bekins^6^). Diatom ooze contains high TOC, and a* can therefore be used as a proxy for TOC. Both TOC and a* show two maxima near 30 mbsf and 100 mbsf, respectively. A third maximum in a* occurs near 180 mbsf, where TOC data are lacking. Even though high TOC contents occur in the top few metres, one should take into account that this part of organic matter decays with a power-law with depth and its impact on the future record is not exactly known. Also, this part of the section still lies in the zone of ongoing pyrite formation.

Based on the observed correlation with TOC it is conceivable that intervals of elevated δ^34^S indeed reflect past episodes of enhanced rates of sulphate-reducing microbial activity. In addition to elevated TOC it is very likely that also the sedimentation rates were larger during these two intervals. Although an age model at sufficiently high resolution is currently not available for these intervals, based on the age model of the last 100 ka at Site 1229 (Contreras *et al*.^7^), higher sedimentation rates co-occur with times of enhanced upwelling on the Peru Margin and accordingly also higher primary productivity, higher organic sedimentation rate and higher TOC in the sediment. Apparently, however, the 100 ka glacial-interglacial cyclicity at Site 1229 has not resulted in elevated δ^34^S throughout the 100-m-thick Pleistocene interval. It is only the large-scale intervals (at 30 and 100 mbsf), which may reflect episodes of enhanced upwelling that lasted long enough to have left a visible imprint in the diagenetic δ^34^S record.

The intervals with elevated δ^34^S also correlate with elevated δ^13^C_DIC_ values in diagenetic dolomite (Fig. 4). There is general consensus that dolomites form in carbonate-free sediment as a result of alkalinity production due to anaerobic metabolic respiration, in particular, sulphate reduction and anaerobic methane oxidation (e.g. Kelts and McKenzie^9^; Baker and Burns^50^; Moore *et al*.^51^, Meister *et al*.^10^). While these processes produce negative δ^13^C_DIC_ values from the decomposition of organic matter, more positive values in the inorganic carbon result from microbial production of isotopically light methane (Claypool and Kaplan^13^). The δ^13^C values in dolomite in the two intervals at 30 and 100 mbsf (Fig. 4, solid diamonds) are far higher than δ^13^C in DIC of the porewater (line), even within the present methanogenic zones. These values can only be explained by strongly enhanced methanogenic activity in the past. This would be consistent with the interpretation of the S isotope record, which also indicates higher microbial activity during these two intervals.

In conclusion, two episodes of enhanced sub-seafloor microbial activity during early and mid Pleistocene are documented independently in the diagenetic S and C isotope records. Apparently the short-term 100 ka cycles described by Contreras *et al*.^7^ are not recorded, but the records are useful for long-term variations in sub-seafloor biosphere activity. In continuous records, a constant ratio of CRS-bound Fe to total Fe provides a useful indicator to exclude that substantial amounts of Fe were re-distributed upon later diagenetic processes and, thus, that the δ^34^S in pyrite represents robust record of microbial activity. Our study thus demonstrates that diagenetic S and C isotope records bear the potential to trace million-year-scale variations in sub-seafloor microbial activity in the deep-time rock record.

## Methods

Five differently reactive iron fractions were sequentially extracted and quantified by the method of Poulton and Canfield^25^ using 5 different solvents: (I) Na-acetate for Fe-carbonates, parts of the acid volatile Fe-sulphides, adsorbed and dissolved Fe, (II) hydroxylamine-HCl for lepidocrocite and ferrihydrite, (III) Na-dithionite for goethite, hematite, and akaganéite, (IV) oxalate for magnetite, and (V) boiling HCl for Fe in sheet silicates. Eluents were analysed by a Thermo iCE 3000 Series atomic absorption mass spectrometer (AAS). The precision of the measurement was better than ±2% (standard deviation). A high extraction efficiency was confirmed by a near 1:1 ratio of total extracted Fe to total Fe from X-ray fluorescence analysis (XRF; Wien *et al*.^52^).

The extraction of acid volatile and chromium reducible S (AVS and CRS) was performed after the standard method of Canfield *et al*.^53^ and Fossing and Jørgensen^31^. CRS-bound Fe was calculated from CRS multiplied by a factor of two, assuming that CRS mainly contains iron sulphide with the stoichiometry FeS_2_ (pyrite). Sulphur isotopes were analysed by the same method as described by Arning *et al*.^54^. Values are reported relative to the Vienna Canyon Diablo Troilite (VCDT) standard.

X-ray absorption near-edge structure (XANES) spectroscopy was carried out at the A1 beamline of the DORIS storage ring at Deutsches Elektronen-Synchrotron (DESY, Hamburg, Germany). Acquisition parameters were described in Meister *et al*.^55^. The samples were prepared as dry pellets or, for the sample at 2.55 mbsf, using anoxically stored wet sediment. In an anaerobic chamber, the sediment was filled into the well in the sample holder, which was tightly sealed with a kapton plate. XANES spectra were collected at the Fe K-edge from 6960 to 8000 eV with 5 eV steps up to 7082 eV and 0.25 eV between 7082 and 7152 eV. A reference foil of metallic Fe(0) was used for internal energy calibration of the monochromator (the first inflection point of the Fe K-edge was set at 7112.1 eV). XANES spectra were processed and analysed using the Horae Athena free software (Newville^56^; Ravel and Newville^57^). Experimental spectra were normalized and fitted to a linear combination of standard spectra of Fe minerals using a least-square minimization procedure.

## Supplementary information

Supplement

Table S1

## References

[1] Parkes RJ (1994). A deep bacterial biosphere in Pacific Ocean sediments. Nature.

[2] D’Hondt, S., Jørgensen, B. B., Miller, J. & ODP Leg 201 Shipboard Scientific Party) Controls on microbial communities in deeply buried sediments, Eastern Equatorial Pacific and Peru Margin, Sites 1225–1231. *Proc. ODP, Init. Repts*. **201**, College Station, TX (Ocean Drilling Program) (2003).

[3] D’Hondt S (2004). Distributions of Microbial Activities in Deep Subseafloor Sediments. Science.

[4] Jørgensen, B. B., D’Hondt, S., Miller, D. J. & Leg 201 Shipboard Scientific Party, Leg 201 Synthesis: Controls on Microbial Communities in Deeply Buried Sediments. *Proc. ODP, Sci. Results* **201** (2006).

[5] Kallmeyer, J. & Wagner, D. Microbial life of the deep biosphere. *Life in Extreme Environments* **1**. Walter De Gruyter GmbH, Berlin/Boston, 325 p. (2014).

[6] Aiello IW, Bekins BA (2010). Milankovitch-scale correlations between deeply buried microbial populations and biogenic ooze lithology. Geology.

[7] Contreras S (2013). Strong glacial-interglacial variation of sub-seafloor microbial activity on the Peruvian shelf. Proc. Natl. Acad. Sci..

[8] Meister P (2015). For the deep biosphere, the present is not always the key to the past: what we can learn from the geological record. Terra Nova, Focus Article.

[9] Kelts, K. & McKenzie, J. A. A comparison of anoxic dolomite from deep-sea sediments: Quaternary Gulf of California and Messinian Tripoli Formation of Sicily. In: *Dolomites of the Monterey Formation and other organic-rich units*(Eds Garrison, R. E., Kastner, M. & Zenger, D. H.), *Pacific Section SEPM* **41**, 19–28 (1984).

[10] Meister P (2007). Dolomite formation in the dynamic deep biosphere: Results from the Peru Margin (ODP Leg 201). Sedimentology.

[11] Schrag DP, Higgins JA, Macdonald FA, Johnston DT (2013). Authigenic carbonate and the history of the global carbon cycle. Science.

[12] Kaplan IR, Rittenberg SC (1964). Microbiological fractionation of sulphur isotopes. J. Gen. Microbiol..

[13] Claypool, C. E. & Kaplan, I. R. The origin and distribution of methane in marine sediments. In: *Natural Gases in Marine Sediments* (Ed. Kaplan, I. R.) *Plenum Press, New York*, 99–140 (1974).

[14] Jørgensen BB (1979). A theoretical model of the stable sulphur isotope distribution in marine sediments. Geochim. Cosmochim. Acta.

[15] Hoefs, J. Stable isotope geochemistry. 8^th^Edition. *Springer Textbooks in Earth Sciences, Geography and Environment*. Springer International Publishing AG, Cham, Switzerland (2018).

[16] Parnell J, Boyce AJ, Hurst A, Davidheiser-Kroll B, Ponicka J (2013). Long term geological record of a global deep subsurface microbial habitat in sand injection complexes. Nature Scientific Reports.

[17] Sim MS, Bosak T, Ono S (2011). Large sulfur isotope fractionation does not require disproportionation. Science.

[18] Hartmann M, Nielsen H (2012). δ^34^S values in recent sea sediments and their significance using several sediment profiles from the western Baltic Sea. Isotopes in environmental and health studies.

[19] Borowski WS, Rodriguez NM, Paull CK, Ussler W (2013). Are ^34^S-enriched authigenic sulfide minerals a proxy for elevated methane flux and gas hydrates in the geologic record?. Marine and Petroleum Geology.

[20] Pellerin A (2018). The sulfur cycle below the sulfate-methane transition of marine sediments. Geochim. Cosmochim. Ac..

[21] Riedinger, N. *et al*. Sulfur cycling in an iron oxide-dominated, dynamic marine depositional system: the Argentine Continental Margin: *Frontiers in Earth Science* **5**, 10.3389/feart.2017.00033 (2017).

[22] Böttcher, M. E. *et al*. Sulfur isotope fractionation by the deep biosphere within sediments of the Eastern Equatorial Pacific and Peru Margin. *Proc. ODP, Sci. Results* **201**, College Station, TX (Ocean Drilling Program) (2006).

[23] Wortmann UG, Bernasconi SM, Böttcher ME (2001). Hypersulfidic deep biosphere indicates extreme sulfur isotope fractionation during single-step microbial sulfate reduction. Geology.

[24] Jørgensen BB, Böttcher ME, Lüschen H, Neretin L, Volkov I (2004). Anaerobic methane oxidation and a seep H_2_S sink generate isotopically heavy sulfides in Black Sea sediments. Geochim. Cosmochim. Acta.

[25] Poulton SW, Canfield DE (2005). Development of a sequential extraction procedure for iron: Implications for iron partitioning in continentally-derived particulates. Chem. Geol..

[26] Berner RA (1970). Sedimentary pyrite formation. Am. J. Sci..

[27] Kasina M (2017). Mineralogical and geochemical analysis of Fe-phases in drill-cores from the Triassic Stuttgart Formation at Ketzin CO_2_ storage site before CO_2_ arrival. Environmental Earth Sciences.

[28] Rennert T, Eusterhues K, De Andrade V, Totsche KU (2012). Iron species in soils on a mofette site studied by Fe K-edge X-ray absorption near-edge spectroscopy. Chem. Geol..

[29] Canfield DE (1989). Reactive iron in marine sediments. Geochim. Cosmochim. Ac..

[30] Mossmann J-R, Aplin AC, Curtis CD, Coleman ML (1990). Sulfur geochemistry at Sites 680 and 686 on the PeruMargin. Proc. ODP, Sci. Results.

[31] Fossing H, Jørgensen BB (1989). Measurement of bacterial sulfate reduction in sediments – evaluation of a single-step chromium reduction method. Biogeochemistry.

[32] Böttcher M, Hetzel A, Brumsack H-J, Schipper A (2006). Sulfur-iron-carbon geochemistry in sediments of the Demarara Rise. Proc. ODP Sci. Results.

[33] Rickard D, Luther GW (2007). Chemistry of iron sulfides. Chem. Rev..

[34] Afonso MDS, Stumm W (1992). Reductive dissolution of iron(III) (hydr)oxides by hydrogen sulfide. Langmuir.

[35] Wächtershäuser G (1988). Pyrite formation, the first energy source for life: a hypothesis. Systematic Applied Microbiology.

[36] Thiel, J., Byrne, J., Kappler, A., Schink B. & Pester M. Pyrite formation from FeS and H_2_S is mediated by a novel type of microbial energy metabolism. *BioRXive*, 10.1101/396978 (2018).

[37] Riedinger N (2005). Diagenetic alteration of magnetic signals by anaerobic oxidation of methane related to a change in sedimentation rate. Geochim. Cosmochim. Acta.

[38] Hartmann M, Nielsen H (2012). δ^34^S-Werte in rezenten Meeressedimenten und ihre Deutung am Beispiel einiger Sedimentprofile aus der westlichen Ostsee. Geologische Rundschau.

[39] Pasquier V (2017). Pyrite sulfur isotopes reveal glacial−interglacial environmental changes. Proc. Natl. Acad. Sci..

[40] Meister P, Liu B, Ferdelman TG, Jørgensen BB, Khalili A (2013). Control of sulphate and methane distributions in marine sediments by organic matter reactivity. Geochim. Cosmochim. Acta..

[41] Chambers LA, Trudinger PA (1979). Microbiological fractionation of stable sulfur isotopes: a review and critique. Geomicrobiology J..

[42] Wing BA, Halevy I (2014). Intracellular metabolite levels shape sulfur isotope fractionation during microbial sulfate respiration. Proc. Natl. Acad. Sci..

[43] Deusner C (2014). Sulfur and oxygen isotope fractionation during sulfate reduction coupled to anaerobic oxidation of methane is dependent on methane concentration. Earth Planet. Sci. Letters.

[44] Rudnicki MD, Elderfield H, Spiro B (2001). Fractionation of sulfur isotopes during bacterial sulfate reduction in deep ocean sediments at elevated temperatures. Geochim. Cosmochim. Ac..

[45] Claypool, G. E. Ventilation of marine sediments indicated by depth profiles of pore water sulfate and δ^34^S. In: Hill, R. J. *et al*. (ed.) Geochemical Investigations in Earth and Space Science: A Tribute to Isaac R. Kaplan. *Geochem. Soc. Spec. Publ*., **9**, 59–65 (2004).

[46] Canfield DE, Thamdrup B (1994). The production of ^34^S-depleted sulfide during bacterial disproportionation of elemental sulfur. Science.

[47] Cypionka H, Smock AM, Böttcher ME (1998). A combined pathway of sulfur compound disproportionation in *Desulfovibrio desulfuricans*. *FEMS Microbiol*. Letters.

[48] Habicht KS, Canfield DE, Rethmeier J (1998). Sulfur isotope fractionation during bacterial reduction and disproportionation of thiosulfate and sulfite. Geochim. Cosmochim. Acta.

[49] Meister, P., Prokopenko, M., Skilbeck, C. G., Watson, M. & McKenzie, J. A. Data report: compilation of total organic and inorganic carbon data from Peru margin and eastern equatorial Pacific drill sites (ODP Legs 112, 138, and 201). *Proc. ODP, Sci. Results* **201** (2005).

[50] Baker PA, Burns SJ (1985). Occurrence and formation of dolomite in organic-rich continental margin sediments. AAPG Bull..

[51] Moore TS, Murray RW, Kurtz AC, Schrag DP (2004). Anaerobic methane oxidation and the formation of dolomite. Earth Planet. Sci. Letters.

[52] Wien K, Wissmann D, Kölling M, Schulz HD (2005). Fast application of X-ray fluorescence spectrometry aboard ship: how good is the new portable Spectro Xepos analyser?. Geo-Mar Lett..

[53] Canfield DE, Raiswell R, Westrich JT, Reaves CM, Berner RA (1986). The use of chromium reduction in the analysis of reduced inorganic sulfur in sediments and shales. Chem. Geol..

[54] Arning E, Birgel D, Brunner B, Peckmann J (2009). Bacterial formation of phosphatic laminites off Peru. Geobiology.

[55] Meister P (2014). Early diagenetic quartz formation at a deep iron oxidation front in the Eastern Equatorial Pacific. Geochim. Cosmochim. Acta.

[56] Newville M (2001). IFFEFIT: interactive XAFS analysis and FEFF fitting. J. Synchrotron Radiat..

[57] Ravel B, Newville M (2005). ATHENA, ARTEMIS, HEPHAESTUS: data analysis for X-ray absorption spectroscopy using IFEFFIT. J. Synchrotron Radiat..

