# Supplementary material for: Sulphur and carbon isotopes as tracers of past sub-seafloor microbial activity
Source: Sci Rep. 2019 Jan 24;9:604. doi: 10.1038/s41598-018-36943-7 (PMC6345876; doi:10.1038/s41598-018-36943-7)
Supplement: Supplementary file 1 — Supplement [file 41598_2018_36943_MOESM1_ESM.pdf]

Supplementary Material

Sulphur and carbon isotopes as tracers of past sub-seafloor microbial activity

Patrick Meister[1], Benjamin Brunner[2], Aude Picard[3], Michael E. Böttcher[4], and Bo Barker Jørgensen[5,6]

[1]Department of Geodynamics and Sedimentology, University of Vienna, Althanstr. 14, 1090 Vienna, Austria, [2]Department of Geological Sciences, University of Texas at El Paso (UTEP), El Paso, TX 85287-1404, U.S.A., [3]Department of Organismic and Evolutionary Biology, Harvard University, 16 Divinity Avenue, Cambridge, MA 02138, U.S.A., [4]Geochemistry & Isotope Biogeochemistry Group, Leibniz-Institute for Baltic Sea Research (IOW), Seestrasse 15, D-18119 Warnemünde, Germany, [5]Center for Geomicrobiology, Aarhus University, Ny Munkegade 114-116, 8000 Aarhus, Denmark, [6]Max-Planck Institute for Marine Microbiology, Celsiusstrasse 1, D-28359 Bremen, Germany.

Correspondence and requests for materials should be addressed to P.M. (patrick.meister@univie.ac.at)

Geological and geochemical setting. ODP Site 1229 is located on the Peruvian shelf, 10 km offshore the coast at a water depth of 150 m (Fig. S1). The sedimentary sequence comprises Pliocene-Pleistocene diatom ooze with variable content of terrigenous material. Due to coastal upwelling, the organic C-content is generally high. However, glacial sealevel lowstands led to a breakdown of the upwelling and caused the oxygen minimum zone to impinge further offshore, such that glacial layers are depleted in organic C (Wefer et al., 1990). In these organic C-poor intercalated glacial layers sulphate reduction rates are likely lower, which may explain why the SMT in the modern porewater is located as deep as 30 mbsf (m below seafloor). In organic C-rich sediments a much shallower SMT would be expected but the SMT may migrate upwards or downwards over time (cf. Contreras et al., 2013).

Contreras, S., Meister, P., Liu, B., Prieto-Mollar, X., Hinrichs, K.-U., Khalili, A., Ferdelman, T.G., Kuypers, M. & Jørgensen, B.B. Strong glacial-interglacial variation of sub-seafloor microbial activity on the Peruvian shelf. *Proc. Natl. Acad. Sci.* **110**, 18098–18103 (2013).

Wefer, G., Heinze, P. & Suess, E. Stratigraphy and sedimentation rates from oxygen isotope composition, organic carbon content, and grain-size distribution at the Peru upwelling region: holes 680B and 686B. In: *Proc. ODP, Sci. Results* (Eds. E. Suess, R. von Huene, et al.), College Station, TX (Ocean Drilling Program), **112**, 355-367 (1990).

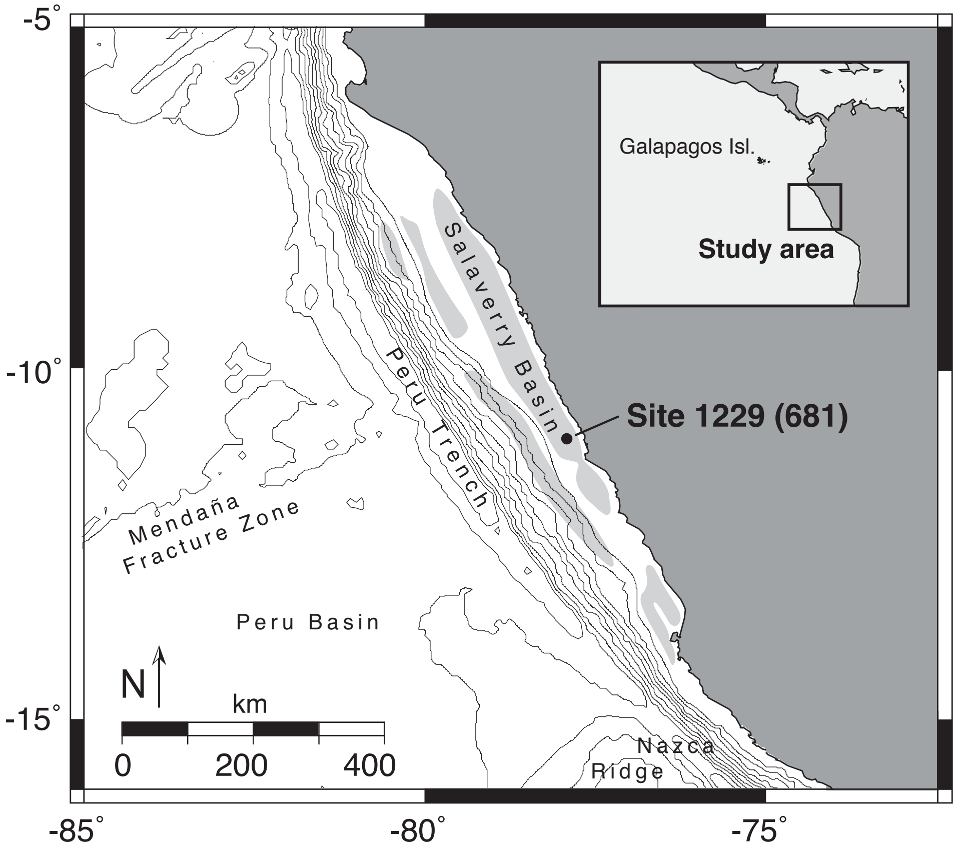

Figure S1 | Map of the Peru Margin showing ODP drill-site 1229.
